# Nut consumption, linoleic and α-linolenic acid intakes, and genetics: how *fatty acid desaturase 1* impacts plasma fatty acids and type 2 diabetes risk in EPIC-InterAct and PREDIMED studies

**DOI:** 10.1186/s12916-025-04187-8

**Published:** 2025-06-09

**Authors:** Susanne Jäger, Olga Kuxhaus, Marcela Prada, Inge Huybrechts, Tammy Y. N. Tong, Nita G. Forouhi, Cristina Razquin, Dolores Corella, Miguel A. Martinez-Gonzalez, Christina C. Dahm, Daniel B. Ibsen, Anne Tjønneland, Jytte Halkjær, Chloé Marques, Claire Cadeau, Xuan Ren, Verena Katzke, Benedetta Bendinelli, Claudia Agnoli, Alberto Catalano, Marta Farràs, Maria-Jose Sánchez, María Dolores Chirlaque López, Marcela Guevara, Dagfinn Aune, Stephen J. Sharp, Nicholas J. Wareham, Matthias B. Schulze

**Affiliations:** 1 Department of Molecular Epidemiology, German Institute of Human Nutrition Potsdam-Rehbruecke, Arthur-Scheunert-Allee 114-116, Nuthetal, 14558 Germany; 2 German Center for Diabetes Research (DZD), Neuherberg, Germany; 3 International Agency for Research on Cancer, World Health Organization, Lyon, France; 4 Nuffield Department of Population Health, Cancer Epidemiology Unit, University of Oxford, Richard Doll Building, Old Road Campus, Oxford, UK; 5 Medical Research Council Epidemiology Unit, University of Cambridge School of Clinical Medicine, Institute of Metabolic Science, Cambridge Biomedical Campus, Cambridge, UK; 6 Department of Preventive Medicine and Public Health, University of Navarra, IdiSNA (Instituto de Investigación Sanitaria de Navarra), Pamplona, Spain; 7 Present Address: CIBER Fisiopatología de la Obesidad y Nutrición (CIBERObn), Instituto de Salud Carlos III, Madrid, Spain; 8 Department of Preventive Medicine and Public Health, School of Medicine, University of Valencia, Valencia, Spain; 9 Department of Public Health, Aarhus University, Aarhus, Denmark; 10 Steno Diabetes Center Aarhus, Aarhus University Hospital, Aarhus, Denmark; 11 Danish Cancer Institute, Diet, Cancer and Health, Copenhagen, DK-2100 Denmark; 12 Department of Public Health, University of Copenhagen, Copenhagen, Denmark; 13 Paris-Saclay University, UVSQ, Inserm, Gustave Roussy, CESP, Villejuif, France; 14 Division of Cancer Epidemiology, German Cancer Research Center, DKFZ, Heidelberg, Germany; 15 Clinical Epidemiology Unit, Institute for Cancer Research, Prevention and Clinical Network (ISPRO), Florence, Italy; 16 Epidemiology and Prevention Unit, Fondazione IRCCS Istituto Nazionale Dei Tumori, Milan, Italy; 17 Department of Translational Medicine, University of Piemonte Orientale, Novara, Italy; 18 Department of Clinical and Biological Sciences, University of Turin, Turin, Orbassano Italy; 19 Unit of Nutrition and Cancer, Epidemiology Research Program, Catalan Institute of Oncology (ICO), Bellvitge Biomedical Research Institute (IDIBELL), L’Hospitalet de Llobregat, 08908 Spain; 20 Escuela Andaluza de Salud Pública (EASP), Granada, 18011 Spain; 21 Instituto de Investigación Biosanitaria Ibs.GRANADA, Granada, 18012 Spain; 22 Centro de Investigación Biomédica en Red de Epidemiología y Salud Pública (CIBERESP), Madrid, 28029 Spain; 23 Department of Epidemiology, IMIB-Arrixaca, Regional Health Council, Murcia University, Murcia, Spain; 24 CIBER in Epidemiology and Public Health (CIBERESP), Madrid, Spain; 25 Instituto de Salud Pública y Laboral de Navarra, Pamplona, 31003 Spain; 26 Navarra Institute for Health Research (IdiSNA), Pamplona, 31008 Spain; 27 Department of Epidemiology and Biostatistics, School of Public Health, Imperial College London, London, UK; 28 Department of Research, Cancer Registry of Norway, Norwegian Institute of Public Health, Oslo, Norway; 29 Department of Nutrition, Oslo New University College, Oslo, Norway; 30 Institute of Nutritional Science, University of Potsdam, Nuthetal, Germany

**Keywords:** Polyunsaturated fatty acids, Fatty acid desaturase, Plasma phospholipid fatty acids, Cohort study, Randomized controlled trial

## Abstract

**Background:**

Dietary guidelines recommend replacing saturated fatty acid with unsaturated fats, particularly polyunsaturated fatty acids. Cohort studies do not suggest a clear benefit of higher intake of polyunsaturated fatty acids but, in contrast, higher circulating linoleic acid (LA) levels—reflective of dietary LA intake, are associated with a reduced risk of type 2 diabetes. However, genetic variants in the *fatty acid desaturase 1* gene (*FADS1*) may influence individual responses to plant-based fats. We explored whether *FADS1* variants influence the relationships of LA and α-linolenic acid (ALA) intakes and nut consumption with plasma phospholipid fatty acid profiles and type 2 diabetes risk in a large-scale cohort study and a randomized controlled trial.

**Methods:**

In the EPIC-InterAct case-cohort (7,498 type 2 diabetes cases, 10,087 subcohort participants), we investigated interactions of dietary and plasma phospholipid fatty acids and nut consumption with *FADS1* rs174547 in relation to incident type 2 diabetes using weighted Cox regression. In PREDIMED (492 participants in the Mediterranean Diet + Nuts intervention group, 436 participants in the control group), we compared changes in plasma phospholipid FAs from baseline to year 1.

**Results:**

In EPIC-InterAct and PREDIMED, nut consumption was positively associated with LA plasma levels and inversely with arachidonic acid, the latter becoming stronger with increasing number of the minor rs174547 C allele (p interaction EPIC-InterAct: 0.030, PREDIMED: 0.003). Although the inverse association of nut consumption with diabetes seemed stronger in participants with rs174547 CC-genotype (HR: 0.73, 95% CI: 0.54–1.00) compared with CT (0.94, 0.81–1.10) or TT (0.90, 0.78–1.05) in EPIC-InterAct, this interaction was not statistically significant.

**Conclusions:**

*FADS1* variation modified the effect of nut consumption on circulating FAs. We did not observe clear evidence that it modified the association between nut consumption and type 2 diabetes risk.

**Supplementary Information:**

The online version contains supplementary material available at 10.1186/s12916-025-04187-8.

## Background

General nutrition guidelines recommend limiting saturated fatty acid (SFA) intake and replacing it with unsaturated fats, particularly polyunsaturated fatty acids (PUFA) [1–3]. Among these, the n-6 PUFA linoleic acid (LA, 18:2n-6) and the n-3 PUFA α-linolenic acid (ALA, 18:3n-3) are essential fatty acids predominantly derived from vegetable oils, nuts, and seeds [4] accounting for the largest proportion of total PUFA intake. Randomized controlled trials (RCTs) have demonstrated that the isocaloric replacement of SFA with PUFA improves glycemic parameters [5]. However, evidence linking plant-derived PUFA and their food sources with type 2 diabetes incidence remains limited and inconclusive [6] and current diabetes-specific guidelines do not recommend plant-derived PUFAs for diabetes prevention, despite their relevance for the nutrition management of diabetes [7, 8].

While cohort studies have examined associations between PUFA intake and type 2 diabetes risk, meta-analyses do not consistently suggest a clear benefit from total PUFA, LA, or ALA intake [9–11]. Similarly, consumption of specific food sources such as nuts has not been clearly linked to lower diabetes risk [10, 12]. In contrast, biomarker-based studies reveal stronger and more consistent associations: higher circulating LA levels—reflective of dietary LA intake [13]—are associated with a reduced risk of type 2 diabetes [9, 14, 15]. Nevertheless, circulating PUFA levels are not solely influenced by dietary intake but are also modulated by metabolic processes, particularly those mediated by genetic variation [6].

Emerging evidence from precision nutrition research highlights the need to identify subgroup-specific effects to refine dietary recommendations [16]. Variants in the *fatty acid desaturase 1* (*FADS1*) and *fatty acid desaturase 2* (*FADS2*) genes, encoding the delta-5 (D5D) and delta-6 desaturases (D6D), respectively, play a critical role in the bioconversion of LA and ALA to longer-chain, highly unsaturated fatty acids (HUFA). These genetic variants have been associated with PUFA blood levels [17, 18] and shown to modify responses to dietary LA and ALA intake, affecting circulating PUFA levels [19–21]. Furthermore, effect modification by *FADS1* variants has been reported for cardiovascular disease (CVD) risk in response to LA biomarkers [22] and ALA intake [23]. However, such gene-diet interactions remain understudied for type 2 diabetes risk. Notably, no interaction was observed for LA biomarkers and type 2 diabetes in pooled cohort studies [15], and the influence of *FADS1* variants on associations between PUFA intake or food sources, such as nuts, with type 2 diabetes risk has not yet been investigated.

To address these gaps, we aim to explore the role of plant-derived PUFA in type 2 diabetes risk through the lens of precision nutrition. Specifically, considering potential interaction with the *FADS1* genotype our objectives is to: 1) analyzed the associations between dietary LA and ALA and diabetes risk, 2) examine the link between plasma LA and ALA levels and diabetes risk, and 3) evaluate the impact of nut consumption, a main source of plant PUFA, on circulating PUFA and diabetes risk.

## Methods

### EPIC-interact case-cohort

#### Study population

The European Prospective Investigation into Cancer and Nutrition (EPIC) cohort study includes ~ 520,000 men and women recruited between 1992–2000 in 23 study centers in 10 European countries. In the majority of study centers, participants were invited from the general adult population residing in a given town or geographical area and being aged 35–70 years [24]. The analytical sample (Additional file 1: Figure S1) was based on the EPIC-InterAct case-cohort [25]. From 340,234 individuals in 8 countries, a subcohort of 16,835 individuals with baseline plasma samples was randomly selected. Additionally, we identified 14,980 verified incident cases of type 2 diabetes until 2007 in EPIC. After exclusions of prevalent diabetes and uncertain diabetes status (*n* = 681), missing genetic data (*n* = 7,870), missing blood fatty acid (FA) data (*n* = 315), missing FA intake (*n* = 95), missing confounder variables (*n* = 634), and data from Sweden due to regional data protection requirements (*n* = 4,314), the analyses were based on 7,498 diabetes cases and 10,087 subcohort participants.

#### Case ascertainment

Incident type 2 diabetes (International Classification of Diseases Tenth Edition [ICD-10] code: E11) was ascertained up to 31 December 2007 by reviewing multiple sources (self-report, linkage to primary and secondary care registers, medication use, hospital admissions, and mortality data) depending on the study center [25]. We sought further evidence for cases with information on incident diabetes from at least one independent source, including individual medical records review in some centers.

#### Nut consumption and FA intake and status assessment

Dietary intake in the 12 months before enrolment was assessed by dietary questionnaires, which varied between countries or study centers [24] and included unspecified nuts and nut spreads, tree nuts, peanuts, or seeds, depending on the center. Considering the different information available from individual questionnaires, we used aggregate intake of nuts (including nut spreads) and seeds as exposure. Nutrient and energy intake has been calculated using the USDA food composition database [26].

FA concentrations in plasma phospholipids, which are considered good biomarkers for dietary intake of LA and ALA [13], were measured in EPIC-InterAct baseline samples as described before [14, 27]. Briefly, the plasma phospholipid fraction was obtained by solid-phase extraction, hydrolyzed and methylated to yield FA methyl esters, which were separated by gas chromatography equipped with flame ionization detection. Samples from cases and subcohort participants were processed in a random order by center, and laboratory staff were blinded to participant characteristics. FAs were identified by their retention times compared with commercial standards and expressed in relative concentration units as a percentage of total phospholipid FAs (mol%). The coefficients of variation were less than 8%, except for ALA (13%).

#### Measurement of covariates

Weight and height were measured with participants not wearing shoes and in light clothing or underwear in the majority of centers, as described previously [28]. Questionnaires assessed demographics, smoking status, physical activity, medical history, and educational level [24]. The diet questionnaires mentioned above also assessed consumption of coffee, tea, fruits, vegetables, and sugar-sweetened beverages.

#### Genetic data and FADS1 SNP selection

Genetic data generation within EPIC-InterAct has been described previously [29]. Briefly, genome-wide genotyping was performed at different times using Illumina 660 W-Quad BeadChip and Illumina HumanCoreExome-12v1 and −24v1 BeadArrays (Illumina, San Diego, CA). The *FADS1/FADS2* gene region is characterized by strong linkage disequilibrium patterns, with a common block including *FADS1* and parts of *FADS2* in European populations [30]. We selected the *FADS1* variant rs174547 (T > C) previously associated with PUFA blood levels (reflecting lower desaturase activity in carriers of the minor C allele), which showed interactions with LA blood levels on CVD risk in an individual-level pooled analysis of 30 cohort studies [22].

### PREDIMED

The PREDIMED (Prevención con Dieta Mediterránea) study is a primary cardiovascular prevention RCT conducted in Spain with 7,447 community-dwelling men (ages 55 to 80 years) and women (60 to 80 years) at high cardiovascular risk (either type 2 diabetes or several cardiovascular risk factors). Methods and design have been reported elsewhere [31, 32]. Briefly, participants were randomly assigned to one of three nutritional interventions: 1) Mediterranean diet (MedDiet) + extra-virgin olive oil (EVOO), 2) MedDiet + nuts, and 3) a control diet (advice to reduce dietary fat).

Within the PREDIMED trial, two nested case-cohort sub-studies were designed to analyze the incidence of CVD and type 2 diabetes. Participants from these two case-cohort studies with available baseline and 1-year samples were selected for the present study (*n* = 1,882). Of these, 1,472 participants had completed baseline and year-1 FA profiling data. The MedDiet + EVOO group (*n* = 529) was excluded given the study focus on nuts. Consequently, the analytical sample included 500 participants from the MedDiet + Nuts group and 443 from the control group. After DNA isolation and quality control exclusions, the final sample included 492 participants in the MedDiet + Nuts group and 436 in the control diet group.

Analyses of plasma phospholipid FA were performed with a method using extraction with tert-butyl methyl ether/methanol, solid phase separation, hydrolysis, and methylation with trimethyl sulfonium hydroxide, and subsequent analysis by gas chromatography [33]. The inter-assay coefficients of variation (*n* = 10) for each plasma FA using flame ionization detection were smaller than 6.4% for all FA. Genotyping of the *FADS1* variant rs174546 (C > T) was performed by real-time PCR followed by fluorescent allelic discrimination. Taqman assays with allele-specific probes were used on the ABI Prism 7900HT Sequence Detection System (Applied Biosystems, Foster City, CA, USA) according to standardized protocols. Since rs174546 (C > T) and rs174547 (T > C) (used in EPIC-InterAct) are in full LD, all analyses refer to rs174547 to be consistent with the results from EPIC-InterAct.

### Statistical analysis

We estimated the correlations between dietary intake of LA and ALA and their plasma phospholipid concentrations in EPIC-InterAct, adjusting for baseline demographic, lifestyle, and dietary characteristics. Country-specific estimates were combined using random effects meta-analysis. Associations of ALA and LA intakes (% of total energy intake) and plasma phospholipid PUFA concentrations (%) with hazard of type 2 diabetes were estimated using Prentice-weighted Cox regression, which accounts for the case-cohort design [34], with PUFAs modeled per 1 SD increment based on the subcohort distribution. The proportional hazards assumption was assessed with Schoenfield residuals. Model 1 was stratified by age (the underlying timescale) and adjusted for sex and center. For FA intake and diabetes risk, model 2 was further adjusted for BMI, smoking, education, physical activity, alcohol, dietary fiber, vitamin C, total energy, carbohydrate, protein, monounsaturated FAs, as well as PUFA minus LA (for LA) or minus ALA (for ALA) (macronutrients expressed as % of total energy intake), thus modeling an isocaloric exchange of SFAs with LA or ALA. We also adjusted this model for consumption of coffee, tea, fruits, vegetables, and sugar-sweetened beverages. In model 3, we replaced PUFA minus LA or ALA with the ratio of n6-PUFA to n3-PUFA intake. For plasma phospholipid FAs and type 2 diabetes risk, model 2 included similar covariates to model 2 for dietary LA and ALA, except we did not include macronutrients. In model 3, we adjusted LA and ALA biomarkers for each other. Model 4 represents model 2 further adjusted for the ratio n6-PUFA/n3-PUFA intake. We also fitted separate models for women and men and estimated country-specific hazard ratios which were pooled using random-effects meta-analysis.

For associations between nut intake and log-transformed (natural log) PUFA plasma phospholipid levels (LA, ALA, γ-linolenic acid [GLA, 18:3n-6], dihomo-γ-linolenic acid [DGLA, 20:3n-6], arachidonic acid [AA, 20:4n-6]), we used linear regression models in the EPIC-InterAct random subcohort adjusted for center, age, sex, BMI, smoking, education, physical activity, alcohol intake, total energy intake, and consumption of coffee, tea, fruits, vegetables, and sugar-sweetened beverages. To estimate the association of nut intake with type 2 diabetes, we used Prentice-weighted Cox regression models stratified by age and adjusted for the same set of variables listed above. Nut consumption was dichotomized into high nut consumers (≥ 4 g/day) and low nut consumers (< 4 g/day), reflecting the non-linear association previously observed in EPIC-InterAct, where no appreciable risk difference was observed between non-consumers and low consumers, while high nut consumers appeared to have a lower risk [35]. We also modeled the effect of adjusting for different plasma phospholipid PUFA.

For interaction analyses, the SNP (rs174547) and a multiplicative interaction term between FA/nut intake or plasma phospholipid FA and SNP were included. The SNP was modeled on an additive scale (as 0, 1 and 2 minor C alleles) and dichotomized as a dominant (CC versus CT + TT) and a recessive model (CC + CT versus TT). We also stratified models by genotype.

In PREDIMED, the effect of the MedDiet + Nuts intervention on PUFA levels, with or without the SNP rs174547 (T > C), was estimated using linear regression adjusted for age, sex, BMI, smoking, and a propensity score that used 30 baseline variables to estimate the probability of assignment to the MedDiet + Nuts or the control group [31]. We compared participants with rs174547 TT- (homozygous for the major allele) vs TC + CC-genotype (heterozygous or homozygous for the minor allele). Desaturase activities were estimated from product/precursor ratios (D6D: ALA/LA; D5D: AA/DGLA).

Analyses in EPIC-InterAct were performed using the Statistical Analysis System (SAS) Enterprise Guide 7.1 with SAS version 9.4 (SAS Institute Inc., Cary, NC, USA). Country estimates were meta-analyzed in R (3.4.3) using the meta R package (version 4.9–0). For data analyses in PREDIMED, we used STATA SE version 16.1 (College Station, TX).

## Results

### Baseline characteristics EPIC-InterAct and PREDIMED

Additional file 1: Table S1 shows baseline characteristics of the EPIC-InterAct case-cohort. Median age and BMI in the random subcohort were approximately 52 years and 26 kg/m^2^, and 37% were male. Individuals with type 2 diabetes were, on average, older and more likely to be male, less educated, and smokers compared to the subcohort. Total PUFA accounted for ~ 6% of total energy intake, and median nut consumption was < 1 g/d. Median LA and ALA levels in plasma phospholipids were 22.7% and 0.3%, respectively, in the EPIC-InterAct subcohort and slightly lower in type 2 diabetes cases.

Characteristics of PREDIMED participants are shown in Additional file 1: Table S2. Mean age of participants was 66 years in the MedDiet + Nuts group and 68 years in the control group and mean BMI was 30 kg/m^2^ in both groups. Similar to EPIC-InterAct, total PUFA contributed ~ 6% to total energy intake and LA levels in plasma phospholipids were 21%.

### Correlation between LA and ALA intake and phospholipid concentrations

In all EPIC-InterAct countries, a weak positive correlation was observed between intake and plasma LA (r: 0.11), whereas no meaningful correlation existed for ALA intake and its plasma concentration (r: 0.02) (Additional file 1: Table S3). Correlation coefficients varied between countries, especially for LA, ranging from 0.05 in France to 0.16 in the UK.

### Association between LA and ALA and risk of type 2 diabetes and interaction with *FADS1* rs174547

Higher LA and ALA intakes were associated with an increased rate of type 2 diabetes (Table 1). In model 2, a 1.5% higher energy contribution from LA (reflecting 1 SD in the subcohort)—isocalorically replacing SFAs—was associated with a 7% higher rate (HR: 1.07, 95% CI: 1.01–1.14). Similarly, a 1 SD (0.15%) higher energy contribution from ALA was associated with a 6% higher rate of type 2 diabetes (1.06, 1.00–1.11). In contrast, LA and ALA plasma phospholipid concentrations were inversely associated with risk of type 2 diabetes. In models adjusting for confounders and mutually adjusting for both PUFA biomarkers (to rule out replacement of other PUFA, similar to model 2 for dietary LA and ALA), HRs for type 2 diabetes for a 1 SD increment in concentrations were 0.82 (95% CI: 0.79–0.85) for LA and 0.95 (0.91–0.99) for ALA. These associations were similar between women and men (Additional file 1: Table S4). Also, excluding cases identified within the first 2 years of follow-up (Additional file 1: Table S5) or further adjusting PUFA intake for trans-FA intake did not substantially affect these estimates. Analyzing associations separate by country (Additional file 1: Figures S3-S5) indicated low heterogeneity, except for plasma ALA, where results from France introduced heterogeneity (no significant heterogeneity after exclusion of France, data not shown).

**Table 1:** Association of dietary linoleic acid (LA) and α-linolenic acid (ALA) intakes and plasma phospholipid fatty acid concentrations with incident type 2 diabetes; EPIC-InterAct study (*n*: 17,128)

Fatty acid	HR (95% CI)			
	Model 1	Model 2	Model 3	Model 4
PUFA intake (% of total energy intake)^a^				
LA	1.07 (1.02; 1.11)	1.07 (1.01; 1.14)	1.07 (1.01; 1.14)	-
ALA	1.07 (1.03; 1.11)	1.06 (1.00; 1.11)	1.07 (1.02; 1.12)	-
PUFA plasma phospholipids (%)^b^				
LA	0.77 (0.74; 0.79)	0.82 (0.78; 0.85)	0.82 (0.79; 0.85)	0.82 (0.78; 0.85)
ALA	0.91 (0.88; 0.94)	0.93 (0.89; 0.98)	0.95 (0.91; 0.99)	0.93 (0.89; 0.98)

Model 1 PUFA intake: stratified by age and adjusted for sex and center

Model 2 PUFA intake: Model 1 with further adjustment for BMI (continuous), smoking (never, former, or current), education (none, primary school, technical or professional school, secondary school, or higher education), physical activity index (inactive, moderately inactive, moderately active, or active), alcohol (none, > 0– < 6, 6– < 12, 12– < 24 and ≥ 24 g/d), consumption of coffee (continuous), tea (continuous), fruits (continuous), vegetables (continuous), and sugar sweetened beverages (continuous), intake of dietary fiber (continuous) and vitamin C (continuous), intake of total energy intake (continuous), carbohydrates, protein, mono-unsaturated fatty acids, as well as polyunsaturated fatty acids minus LA (for LA) or minus ALA (for ALA) (all expressed as E%)

Model 3 PUFA intake: Model 1 with further adjustment for BMI (continuous), smoking (never, former, or current), education (none, primary school, technical or professional school, secondary school, or higher education), physical activity index (inactive, moderately inactive, moderately active, or active), alcohol (none, > 0– < 6, 6– < 12, 12– < 24 and ≥ 24 g/d), consumption of coffee (continuous), tea (continuous), fruits (continuous), vegetables (continuous), sugar sweetened beverages (continuous), intake of dietary fiber (continuous) and vitamin C (continuous), intake of total energy intake (continuous), carbohydrates, protein, mono-unsaturated fatty acids (all expressed as E%), and ratio of n-6:n-3 PUFA intake

Model 1 PUFA plasma phospholipids: stratified by age and adjusted for sex and center

Model 2 PUFA plasma phospholipids: Model 1 with further adjustment for BMI (continuous), smoking (never, former, or current), education (none, primary school, technical or professional school, secondary school, or higher education), physical activity index (inactive, moderately inactive, moderately active, or active), alcohol (none, > 0– < 6, 6– < 12, 12– < 24 and ≥ 24 g/d), intake of dietary fiber (continuous) and vitamin C (continuous), intake of total energy intake (continuous), consumption of coffee (continuous), tea (continuous), fruits (continuous), vegetables (continuous), and sugar-sweetened beverages (continuous)

Model 3 PUFA plasma phospholipids: Model 2 + mutual adjustment of LA/ALA biomarkers

Model 4 PUFA plasma phospholipids: Model 2 + ratio of n-6:n-3 PUFA intake

^a^HRs per 1 SD of LA/ALA intake (per 1.5% of total energy intake from dietary LA / per 0.15% of total energy intake from dietary ALA)

^b^HRs per 1 SD of LA/ALA biomarker concentration

Genotype distributions for *FADS1* rs174547 were relatively homogenous across EPIC-InterAct countries (Additional file 1: Table S6). The minor C allele had a frequency of ~ 32%. We observed no evidence that the associations of dietary LA and ALA or their plasma phospholipid levels with type 2 diabetes risk differed between *FADS1* rs174547 genotypes (all *p* values > 0.14) (Table 2).

**Table 2:** Interaction of linoleic acid (LA) and α-linolenic acid (ALA) intake and plasma phospholipid concentrations with rs174547 in the *FADS1* gene for the risk of type 2 diabetes, EPIC-InterAct study (*n*: 17,128)

	**HR (95% CI)**			**P interaction**		
	**Genotype *FADS1* rs174547**					
	**TT**	**CT**	**CC**	**additive**^a^	**dominant**^b^	**recessive**^c^
N	8,007	7,340	1,781	17,128	17,128	17,128
N cases	3,505	3,231	762	7,498	7,498	7,498
PUFA intake (% of total energy intake)^d^						
LA	1.10 (1.01; 1.20)	1.06 (0.98; 1.15)	1.15 (0.95; 1.38)	0.167	0.145	0.559
ALA	1.07 (0.99; 1.16)	1.06 (0.99; 1.14)	1.11 (0.94; 1.30)	0.210	0.162	0.722
PUFA plasma phospholipids (%)^e^						
LA	0.84 (0.79; 0.90)	0.78 (0.73; 0.83)	0.82 (0.72; 0.92)	0.576	0.402	0.848
ALA	0.93 (0.87; 0.98)	0.95 (0.89; 1.02)	0.94 (0.83; 1.07)	0.241	0.262	0.584

^a^considering multiplicative interaction, SNP is coded per C allele (0,1,2)

^b^considering multiplicative interaction, SNP coding: TT = 0, CT + CC = 1

^c^considering multiplicative interaction, SNP coding: TT + TC = 0, CC = 1

^d^HRs per 1 SD (per 1.5% of total energy intake from dietary LA / per 0.15% of total energy intake from dietary ALA); models were stratified by age and adjusted for sex, center, BMI (continuous), smoking (never, former, or current), education (none, primary school, technical or professional school, secondary school, or higher education), physical activity index (inactive, moderately inactive, moderately active, or active), alcohol (none, > 0– < 6, 6– < 12, 12– < 24 and ≥ 24 g/d), intake of dietary fiber (continuous) and vitamin C (continuous), intake of total energy intake (continuous), carbohydrates, protein, mono-unsaturated fatty acids, as well as polyunsaturated fatty acids minus LA (for LA) or minus ALA (for ALA) (all expressed as E%), consumption of coffee (continuous), tea (continuous), fruits (continuous), vegetables (continuous), and sugar-sweetened beverages (continuous)

^e^HRs per 1 SD of LA/ALA biomarker concentration; models were stratified by age and adjusted for sex, center, BMI (continuous), smoking (never, former, or current), education (none, primary school, technical or professional school, secondary school, or higher education), physical activity index (inactive, moderately inactive, moderately active, or active), alcohol (none, > 0– < 6, 6– < 12, 12– < 24 and ≥ 24 g/d), intake of dietary fiber (continuous) and vitamin C (continuous), intake of total energy intake (continuous), consumption of coffee (continuous), tea (continuous), fruits (continuous), vegetables (continuous), and sugar-sweetened beverages (continuous)

### Association between nut consumption and plasma phospholipid FAs

In the EPIC-InterAct random subcohort, nut consumption was positively associated with plasma phospholipid LA levels and inversely associated with DGLA and AA (Additional file 1: Table S7). Notably, a stronger inverse association with AA was observed with higher numbers of the minor *FADS1* rs174547 C-allele (p interaction additive model: 0.030).

**Fig. 1:**
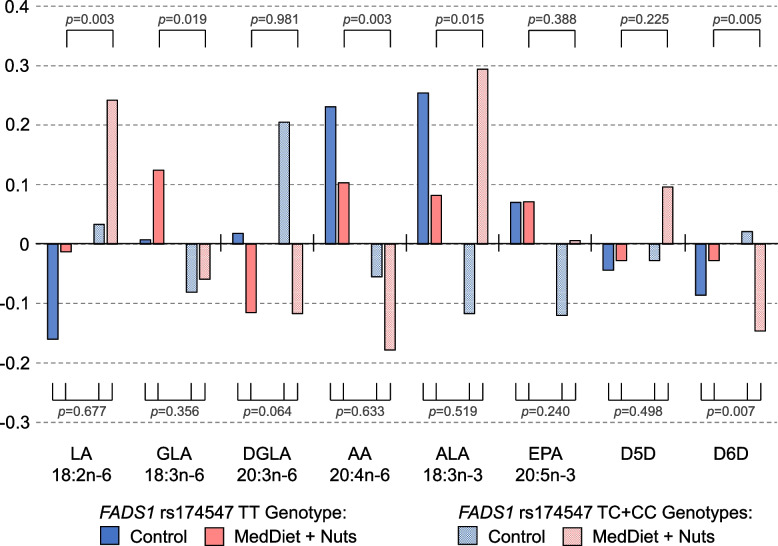
Mean adjusted* one-year changes in *n*−6 and *n*−3 PUFA depending on the intervention group and the *FADS1* rs174547 (T > C) genotype, PREDIMED trial. *Adjusted for age, sex, baseline BMI, smoking and a propensity score that used 30 baseline variables to estimate the probability of assignment to the MedDiet + Nuts or to the control group. One-year changes are expressed as SD changes. *P* values refer to a comparison between TT and TC + CC genotype subgroups (dominant model) within the MedDiet + Nuts group and to the interaction between intervention groups and genotype. AA – arachidonic acid, ALA—α-linolenic acid, D5D – estimated delta-5 desaturase activity, D6D – estimated delta-6 desaturase activity, DGLA—dihomo-γ-linolenic acid, EPA – eicosapentanoic acid, GLA—γ-linolenic acid, LA – linoleic acid

In the PREDIMED RCT, the MedDiet + Nuts increased plasma phospholipids LA and ALA levels more compared to control over one year of intervention. These increases were substantially larger in participants carrying the *FADS1* rs174547 C-allele (CT or CC-genotype) compared to those with TT-genotype (Fig. 1): the mean adjusted change of LA was −0.01 SD in MedDiet + Nuts participants with TT-genotype, while it was + 0.24 SD in participants with TC or CC-genotype (*p* = 0.003). For ALA, the changes were + 0.08 SD versus + 0.29 SD, respectively (*p* = 0.015). Similar to EPIC-InterAct, changes in AA with higher nut consumption in the MedDiet + Nuts group depended on *FADS1* rs174547 genotype (increase in TT genotype, decrease in C-allele carriers; *p* = 0.003). Estimated D6D activity declined with higher nut consumption in the MedDiet + Nuts group more strongly in participants carrying the minor rs174547 C-allele compared to those with TT-genotype, with more pronounced changes in the MedDiet + Nuts group than the control group (*p* interaction = 0.007).

### Nut consumption and risk of type 2 diabetes and interaction with *FADS1* rs174547

The HR comparing participants who consumed at least 4 g/d of nuts with those who consumed less was 0.90 (95% CI 0.81–1.00, *p* = 0.054) in EPIC-InterAct (Table 3). This association appeared to be stronger in participants with the *FADS1* rs174547 CC-genotype (HR: 0.73, 95% CI: 0.54–1.00) compared with CT (0.94, 0.81–1.10) or TT (0.90, 0.90, 0.78–1.05), however, none of the p-values for interaction were significant. Adjustment for LA levels attenuated the associations across all rs174547 genotype strata, while adjustment for AA slightly affected the association in participants with CC-genotype (HR: 0.71, 95% CI: 0.52–0.97).

**Table 3:** Association between consumption of nuts and incident type 2 diabetes and interaction with rs174547 in the *FADS1* gene, EPIC-InterAct study (*n*: 17,128)

	**HR**^a^ **(95% CI) for type 2 diabetes**				**P interaction**		
	**Genotype *FADS1* rs174547**						
	**All**	**CC**	**CT**	**TT**	**additive**^**b**^	**dominant**^**c**^	**recessive**^**d**^
Nut consumption	0.90 (0.81; 1.00)	0.73 (0.54; 1.00)	0.94 (0.81; 1.10)	0.90 (0.78; 1.05)	0.638	0.964	0.304
+ plasma LA	0.93 (0.84; 1.03)	0.74 (0.54; 1.01)	0.97 (0.84; 1.13)	0.92 (0.79; 1.07)	-	-	-
+ plasma ALA	0.90 (0.81; 1.00)	0.74 (0.54; 1.00)	0.94 (0.81; 1.10)	0.90 (0.77; 1.04)	-	-	-
+ plasma GLA	0.91 (0.82; 1.01)	0.72 (0.53; 0.99)	0.95 (0.81; 1.11)	0.91 (0.79; 1.06)	-	-	-
+ plasma DGLA	0.92 (0.83; 1.01)	0.73 (0.53; 0.99)	0.95 (0.81; 1.10)	0.91 (0.78; 1.06)	-	-	-
+ plasma AA	0.90 (0.81; 1.00)	0.71 (0.52; 0.97)	0.94 (0.81; 1.10)	0.90 (0.78; 1.05)	-	-	-

AA – arachidonic acid, ALA—α-linolenic acid, DGLA – dihomo-γ-linolenic acid, GLA—γ-linolenic acid, LA—linoleic acid

^a^HRs refer to a comparison of participants consuming ≥ 4 g/d with those consuming less. Models were stratified for age and adjusted for sex, center, BMI (continuous), smoking (never, former, or current), education (none, primary school, technical or professional school, secondary school, or higher education), physical activity index (inactive, moderately inactive, moderately active, or active), alcohol (none, > 0– < 6, 6– < 12, 12– < 24 and ≥ 24 g/d), intake of total energy intake (continuous), consumption of coffee (continuous), tea (continuous), fruits (continuous), vegetables (continuous), and sugar-sweetened beverages (continuous)

^b^considering multiplicative interaction between nut consumption and *FADS1* rs174547 genotype; SNP is coded per C allele, additive model (0,1,2)

^c^considering multiplicative interaction between nut consumption and *FADS1* rs174547 genotype; SNP coding: TT = 0, CT + CC = 1

^d^considering multiplicative interaction between nut consumption and *FADS1* rs174547 genotype; SNP coding: TT + TC = 0, CC = 1

## Discussion

Nut consumption was associated with higher LA levels in EPIC-InterAct and LA and ALA levels increased in response to a Mediterranean diet supplemented with nuts in PREDIMED. The effect of nut consumption on LA, ALA, and downstream AA depended on the *FADS1* rs174547 genotype. However, we did not observe a statistically significant interaction of nut consumption with *FADS1* in relation to type 2 diabetes risk. We observed that LA and ALA intake was associated with a higher risk of type 2 diabetes, but in contrast, plasma concentrations were associated with lower risk in EPIC-Interact.

Our results, from the observational EPIC-InterAct study and the PREDIMED trial, show that while nut consumption increases circulating LA and ALA levels, the effect on downstream n-6 PUFA (AA) levels depends on *FADS1*. These findings align with previous RCTs on PUFA supplementation and genetic variance in *FADS1* [19–21]. The C-allele of *FADS1* rs174547 relates to a haplotype associated with a limited capacity to synthesize HUFA, which is very common in several Indigenous American populations, rare in populations of African descent, and moderately prevalent in Europeans [6]. The consequences of a different genetic make-up for producing HUFA from LA and ALA for cardiometabolic health are still debated. It has been argued that the *FADS1/2* haplotype associated with efficient conversion of dietary LA and ALA to HUFA (corresponding to the T allele of rs174547) may cause an imbalance between plasma n-6 and n-3 HUFA and increase pro-inflammatory eicosanoids production from AA at high dietary LA intake [36]. However, following a high-LA diet in the FADSDIET and FADSDIET2 RCTs, high sensitive C-reactive protein levels decreased in individuals with the *FADS1* rs174550 TT-genotype (efficient conversion), whereas levels increased or remained unchanged in individuals with the CC-genotype (p interaction < 0.05) [20, 21]. Thus, a genetic make-up supporting LA and ALA bioconversion to HUFA may reduce the need for intake of n-3 HUFA, and high LA intake in the context of high bioconversion capacity would rather be beneficial [6]. Still, we did not detect a statistically significant interaction of nuts consumption, nor of LA/ALA intake and plasma phospholipid levels, and *FADS1* in relation to diabetes risk. Similarly, in a pooled analysis of cohort studies, no interaction was observed for LA biomarkers [15]. In contrast, interactions of PUFA status [22] or intake [23] with *FADS1/2* variants have been reported for CVD.

Our observation that higher dietary intakes of LA and ALA at the expense of SFA were related to a modestly increased risk of type 2 diabetes was unexpected, given results of previous cohort studies [9, 10]. In particular, our findings contrast with those from three pooled large US cohorts, where isocaloric replacement of SFAs with LA (5% energy) was associated with a 14% lower diabetes risk [37]. In addition, meta-analyses of cohort studies suggest that higher ALA intakes are not associated with risk of type 2 diabetes [10, 38, 39]. Similarly, post-hoc analyses of two RCTs do not support an increased risk of type 2 diabetes with higher ALA intake (RR 0.66, 95% CI: 0.33–1.39) [40]. An increased risk with higher LA and ALA intake is also biologically implausible, as RCTs on glucose-related traits have not shown adverse effects from higher intake [5, 40]. LA and ALA intake estimates were based on combining EPIC food consumption data with the USDA food composition database, and might involve misclassification errors. We observed only weak or near-zero correlations between LA and ALA intakes and plasma phospholipid levels, considered biomarkers of intake of these PUFA [13, 41]. A previous comparison in EPIC, based on different participants, also observed poor correlations between LA/ALA intake and biomarker levels, unlike moderate to high correlations observed for other FA (e.g. trans-FAs, long-chain n-3 PUFA) [26]. Noteworthy, the associations between estimated intakes and diabetes risk differ substantially from those of PUFA biomarkers. Our observation that LA and ALA plasma phospholipid levels are associated with lower risk of type 2 diabetes has been described previously from EPIC-InterAct [14] and is consistent with evidence from similar cohort studies [9, 15, 39]. Given these arguments, our observation for dietary LA and ALA should be considered with caution.

Our study has several limitations. We selected a single SNP within the *FADS1/FADS2* region, despite other SNPs being associated with PUFA concentrations [17]; however, rs174547 is in perfect linkage disequilibrium with rs174546 (R^2^ = 1.0, D’ = 1.0), which shows a significant interaction with PUFA intake on CVD [23], and rs174547 was previously investigated in a large pooled analysis of cohort studies on LA biomarkers and type 2 diabetes risk [15]. Rs174546, identified as the functional variant within the *FADS1* cluster altering miRNA binding sites in the *FADS1* 3′UTR [42], belongs to the 10 percent most deleterious variants within the human genome [43]. Furthermore, we only considered nuts as a dietary source of LA and ALA. While higher nut consumption was associated with a lower risk of type 2 diabetes in EPIC-InterAct, other LA and ALA sources (margarine, vegetable oils) were not appreciably associated [35]. We also considered overall consumption of nuts and seeds, although their composition, association with type 2 diabetes risk, and potential for interaction with *FADS1/2* genotypes may vary within this group. A decreased risk of type 2 diabetes associated with high consumption of nuts likely goes beyond the contribution of PUFA, e.g. could involve high content of fiber, magnesium, or polyphenols and other bioactive compounds. Furthermore, we only considered plasma phospholipid PUFAs, while more comprehensive lipid profiling likely better captures dietary exchanges of SFAs with PUFAs [44]. As with observational studies in general, residual confounding may explain the observed associations in EPIC-Interact. However, our finding of an interaction between *FADS1* and nut consumption in EPIC-InterAct was complemented by data from a large RCT, PREDIMED. Although EPIC-InterAct is a large study, the low frequency of the rs174547 CC-genotype and measurement error in dietary exposures probably may have limited the statistical power to detect interactions. The studied populations were almost exclusively of European descent, which limits generalisability to other populations.

## Conclusions

Genetic variation in *FADS1* modifies the effect of nut consumption on circulating PUFAs. However, we did not observe clear evidence that this variation modifies the association of nut consumption with type 2 diabetes risk. Further studies on PUFA intake or their major food sources and studies considering other cardiometabolic outcomes are needed to clarify the importance of *FADS1/2* variation for precision prevention strategies.

## Supplementary Information

Additional file 1: Supplementary tables and figures. Figure S1. Flow chart of participants of the EPIC-InterAct case-cohort. Figure S2. Association of dietary linoleic acid with incident type 2 diabetes by country in EPIC-InterAct. Figure S3. Association of dietary α-linolenic acid with incident type 2 diabetes by country in EPIC-InterAct. Figure S4. Association of plasma phospholipid linoleic acid with incident type 2 diabetes by country in EPIC-InterAct. Figure S5. Association of plasma phospholipid α-linolenic acid with incident type 2 diabetes by country in EPIC-InterAct. Table S1. Baseline characteristics of the EPIC-InterAct case-cohort. Table S2. Baseline characteristics of the PREDIMED study. Table S3: Partial Spearman correlations between diet and plasma linoleic acid and α-linolenic acidin EPIC-InterAct. Table S4. Pooled association between dietary linoleic acid and α-linolenic acid intakes and plasma biomarkers and type 2 diabetes by sex in EPIC-InterAct. Table S5. Association of dietary linoleic acid and α-linolenic acid intakes and plasma fatty acid biomarkers with incident type 2 diabetes after exclusion of cases identified within the first 2 years of follow-up. Table S6. Genotype distribution of *FADS1* rs174547by country in EPIC-InterAct. Table S7. Association between consumption of nuts and plasma phospholipid fatty acids and interaction with *FADS1* rs174547.

## Data Availability

EPIC data and biospecimens are available for investigators who seek to answer important questions on health and disease in the context of research projects that are consistent with the legal and ethical standard practices of the International Agency for Research on Cancer (IARC), WHO, and the EPIC centres. The primary responsibility for accessing the data, obtained in the frame of the present publication, belongs to the EPIC centres that provided them. Access to EPIC data can be requested to the EPIC Steering Committee, as detailed in the EPIC-Europe Access Policy. Investigators of PREDIMED will be happy to provide access to the PREDIMED dataset (including data dictionaries), making possible the replication of the main analyses used for the present article. Due to the restrictions imposed by the Informed Consent and the Institutional Review Board, bona fide investigators interested in analyzing the PREDIMED dataset used for the present article may submit a brief proposal and statistical analysis plan to the PREDIMED Steering Committee. Upon approval from the PREDIMED Steering Committee and Institutional Review Boards, the data will be made available to them using an onsite secure access data enclave.

## Abbreviations

AA: Arachidonic acid
ALA: α-Linolenic acid
BMI: Body mass index
CVD: Cardiovascular disease
DGLA: Dihomo-γ-linolenic acid
D5D: Delta-5 desaturase
D6D: Delta-6 desaturase
EPIC: European Prospective Investigation into Cancer and Nutrition
FA: Fatty acid
FADS1: *Fatty acid desaturase 1*
FADS 2: *Fatty acid desaturase 2*
GLA: γ-Linolenic acid
HUFA: Highly unsaturated fatty acids
ICD: International Classification of Diseases
LA: Linoleic acid
MedDiet: Mediterranean diet
PREDIMED: Prevención con Dieta Mediterránea
PUFA: Polyunsaturated fatty acids
RCT: Randomized controlled trial
SFA: Saturated fatty acid
