# Supplementary material for: Nut consumption, linoleic and α-linolenic acid intakes, and genetics: how fatty acid desaturase 1 impacts plasma fatty acids and type 2 diabetes risk in EPIC-InterAct and PREDIMED studies
Source: BMC Med. 2025 Jun 9;23:344. doi: 10.1186/s12916-025-04187-8 (PMC12147277; doi:10.1186/s12916-025-04187-8)
Supplement: Supplementary file 1 — Additional file 1: Supplementary tables and figures. Figure S1. Flow chart of participants of the EPIC-InterAct case-cohort. Figure S2. Association of dietary linoleic acid with incident type 2 diabetes by country in EPIC-InterAct. Figure S3. Association of dietary α-linolenic acid with incident type 2 diabetes by country in EPIC-InterAct. Figure S4. Association of plasma phospholipid linoleic acid with incident type 2 diabetes by country in EPIC-InterAct. Figure S5. Association of plasma phospholipid α-linolenic acid with incident type 2 diabetes by country in EPIC-InterAct. Table S1. Baseline characteristics of the EPIC-InterAct case-cohort. Table S2. Baseline characteristics of the PREDIMED study. Table S3: Partial Spearman correlations between diet and plasma linoleic acid and α-linolenic acidin EPIC-InterAct. Table S4. Pooled association between dietary linoleic acid and α-linolenic acid intakes and plasma biomarkers and type 2 diabetes by sex in EPIC-InterAct. Table S5. Association of dietary linoleic acid and α-linolenic acid intakes and plasma fatty acid biomarkers with incident type 2 diabetes after exclusion of cases identified within the first 2 years of follow-up. Table S6. Genotype distribution of FADS1 rs174547by country in EPIC-InterAct. Table S7. Association between consumption of nuts and plasma phospholipid fatty acids and interaction with FADS1 rs174547. [file 12916_2025_4187_MOESM1_ESM.docx]

* The sub-cohort is a random selection of a subset of the full cohort at baseline. It therefore includes a proportion of incident cases identified in the full cohort (proportional to the sampling fraction).

**Exclusions:**

- Missing SNPs (rs174546 rs174547): n=7,870
- Missing blood fatty acids: n=315
- Missing fatty acid intake: n=95
- Missing covariables: n=634

**Exclusions:**

- Participants from Sweden: n=4,314

**Exclusions:**

- prevalent or uncertain diabetes status: n=681

Overlap: 457 participants with type 2 diabetes

Overlap: 585 participants with type 2 diabetes

Overlap*: 778 participants with type 2 diabetes

Overlap: 778 participants with type 2 diabetes

**Random sub-cohort:**

16,835 participants

**Verified incident type 2 diabetes in full cohort:**

14,980 participants

**Verified incident type 2 diabetes:**

14,980 participants

**Random sub-cohort:**

16,154 participants

**Verified incident type 2 diabetes:**

9,548 participants

**Random sub-cohort:**

12,479 participants

**Verified incident type 2 diabetes:**

7,498 participants

**Random sub-cohort:**

10,087 participants

**Figure S1.** Flow chart of participants, EPIC-InterAct case-cohort


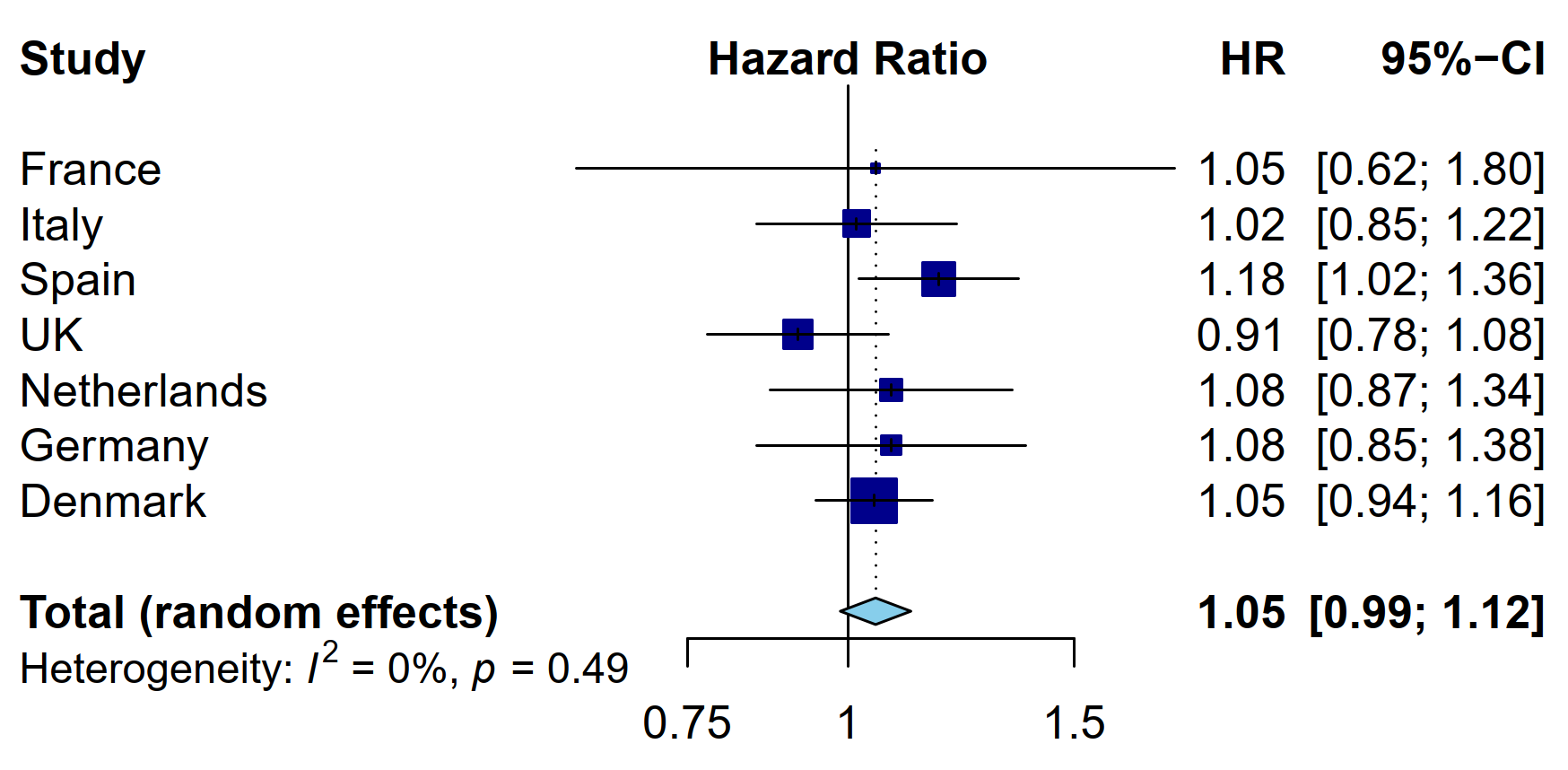


**Figure S2.** Association of dietary linoleic acid with incident type 2 diabetes by country; EPIC-InterAct study

HRs per 1 SD (1.5% of total energy intake) of linoleic acid intake. Model stratified by age and adjusted for sex, center, BMI (continuous), smoking (never, former, or current), education (none, primary school, technical or professional school, secondary school, or higher education), physical activity index (inactive, moderately inactive, moderately active, or active), alcohol (none, >0–<6, 6–<12, 12–<24 and ≥24 g/d), consumption of coffee (continuous), tea (continuous), fruits (continuous), vegetables (continuous), and sugar sweetened beverages (continuous), intake of dietary fiber (continuous) and vitamin C (continuous), intake of total energy intake (continuous), carbohydrates, protein, mono-unsaturated fatty acids, as well as polyunsaturated fatty acids minus LA (all expressed as E%). Country-specific hazard ratios were pooled using random-effects meta-analysis.


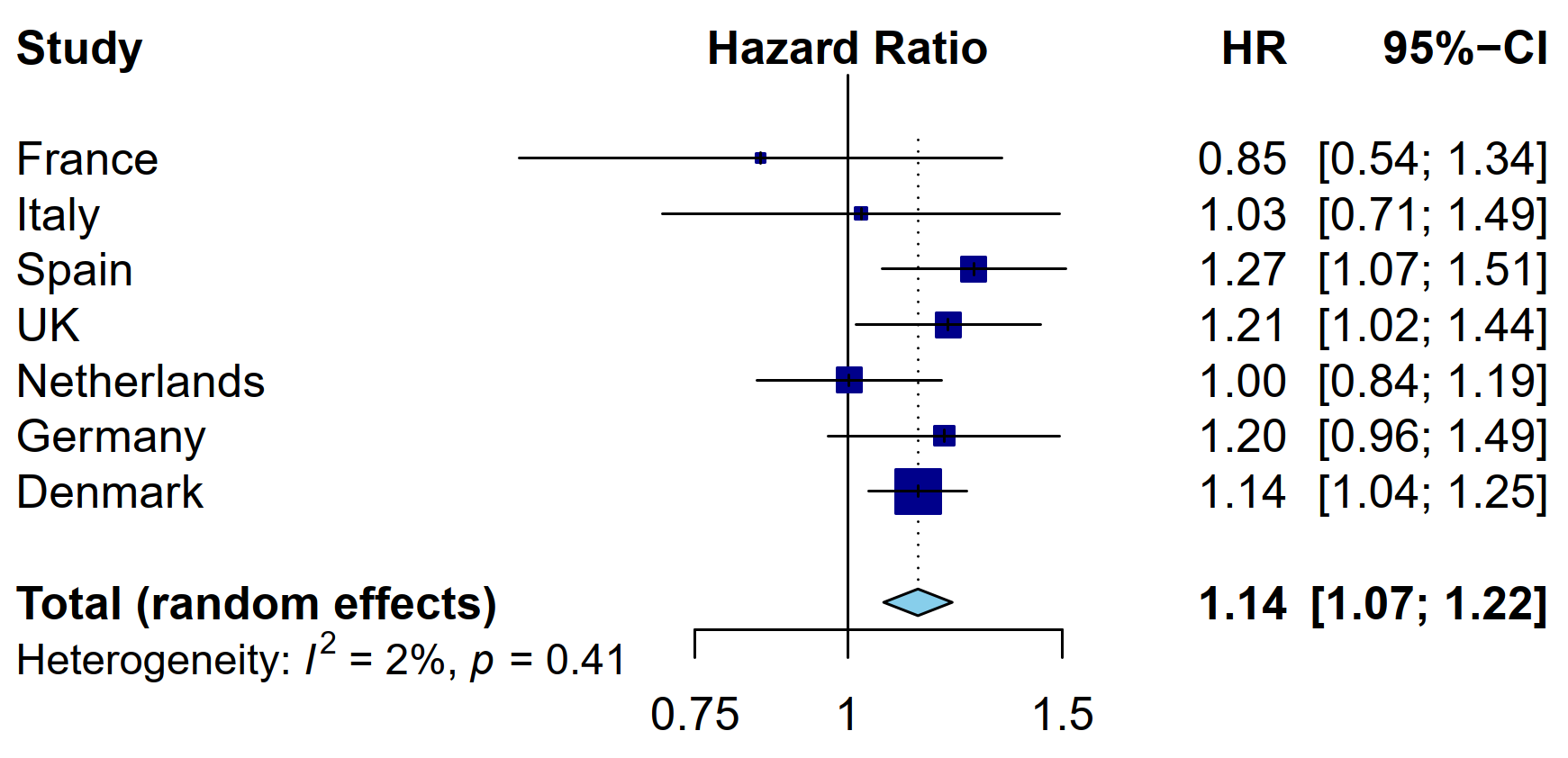


**Figure S3.** Association of dietary α-linolenic acid with incident type 2 diabetes by country; EPIC-InterAct study

HRs per 1 SD (0.15% of total energy intake) of α-linolenic acid intake. Model stratified by age and adjusted for sex, center, BMI (continuous), smoking (never, former, or current), education (none, primary school, technical or professional school, secondary school, or higher education), physical activity index (inactive, moderately inactive, moderately active, or active), alcohol (none, >0–<6, 6–<12, 12–<24 and ≥24 g/d), consumption of coffee (continuous), tea (continuous), fruits (continuous), vegetables (continuous), and sugar sweetened beverages (continuous), intake of dietary fiber (continuous) and vitamin C (continuous), intake of total energy intake (continuous), carbohydrates, protein, mono-unsaturated fatty acids, as well as polyunsaturated fatty acids minus ALA (all expressed as E%). Country-specific hazard ratios were pooled using random-effects meta-analysis.


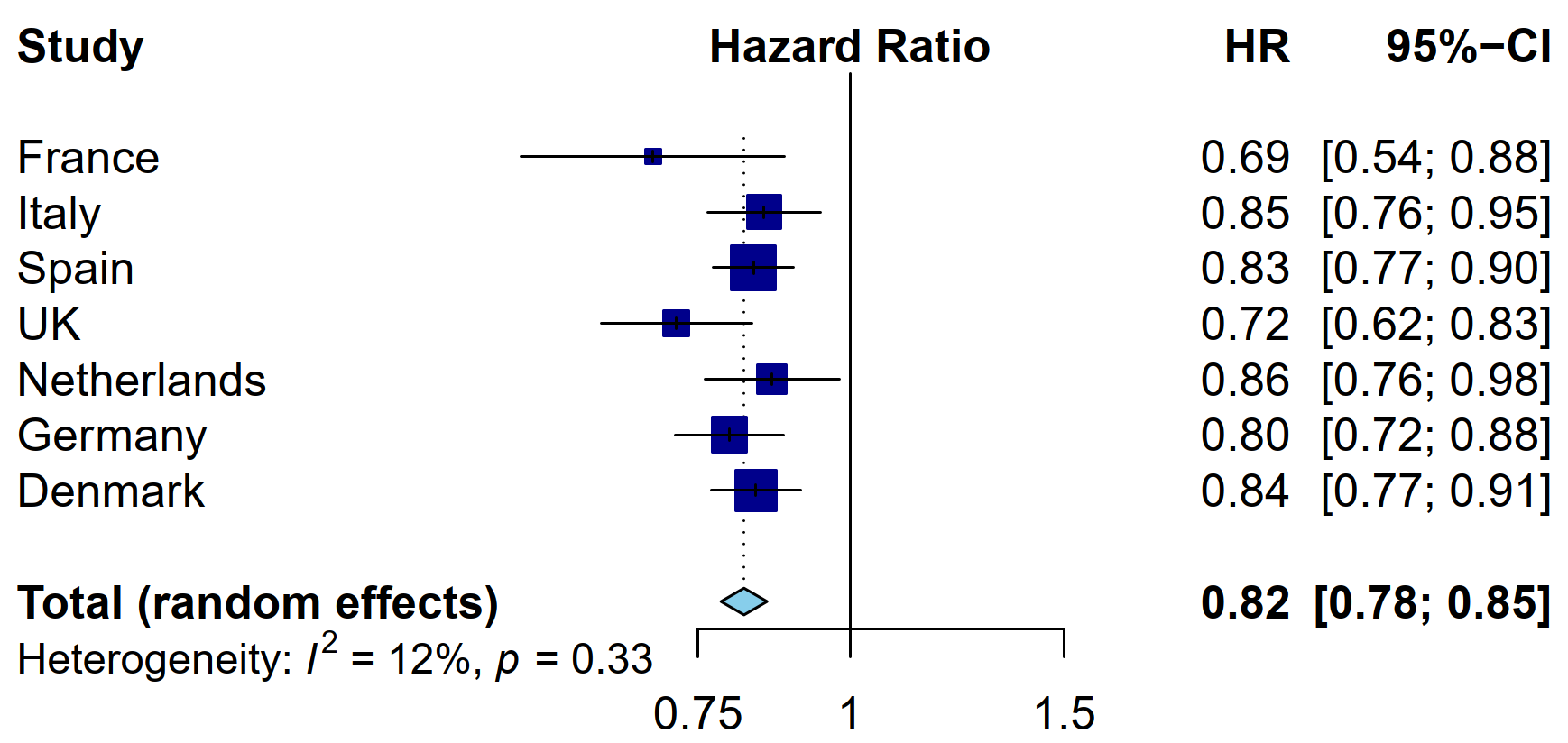


**Figure S4.** Association of plasma phospholipid linoleic acid with incident type 2 diabetes by country; EPIC-InterAct study

HRs per 1 SD of linoleic acid biomarker concentration. Model stratified by age and adjusted for sex, center, BMI (continuous), smoking (never, former, or current), education (none, primary school, technical or professional school, secondary school, or higher education), physical activity index (inactive, moderately inactive, moderately active, or active), alcohol (none, >0–<6, 6–<12, 12–<24 and ≥24 g/d), intake of dietary fiber (continuous) and vitamin C (continuous), intake of total energy intake (continuous), consumption of coffee (continuous), tea (continuous), fruits (continuous), vegetables (continuous), and sugar-sweetened beverages (continuous). Country-specific hazard ratios were pooled using random-effects meta-analysis.


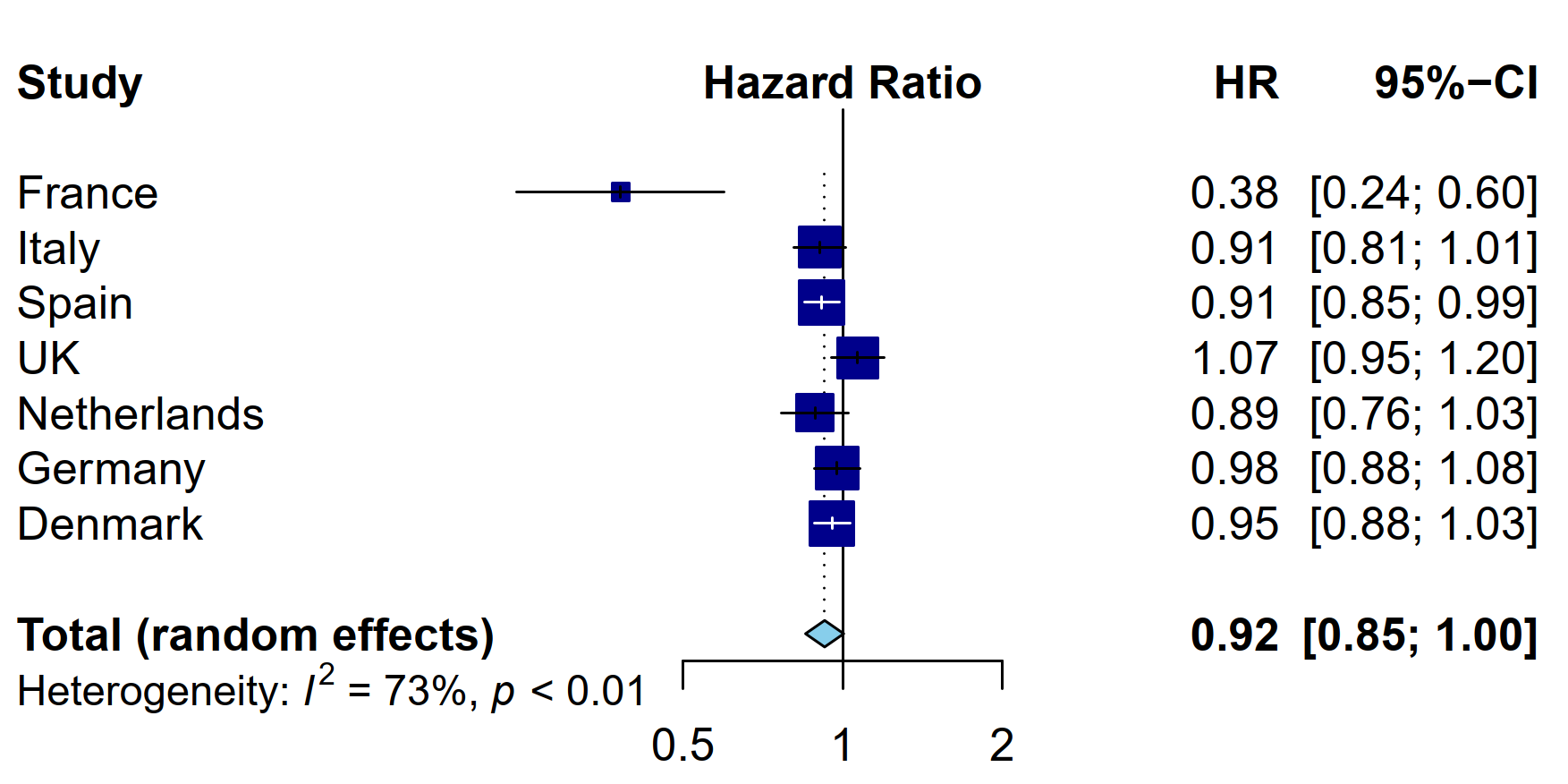


**Figure S5.** Association of plasma phospholipid α-linolenic acid with incident type 2 diabetes by country; EPIC-InterAct study

HRs per 1 SD of α-linolenic acid biomarker concentration. Model stratified by age and adjusted for sex, center, BMI (continuous), smoking (never, former, or current), education (none, primary school, technical or professional school, secondary school, or higher education), physical activity index (inactive, moderately inactive, moderately active, or active), alcohol (none, >0–<6, 6–<12, 12–<24 and ≥24 g/d), intake of dietary fiber (continuous) and vitamin C (continuous), intake of total energy intake (continuous), consumption of coffee (continuous), tea (continuous), fruits (continuous), vegetables (continuous), and sugar-sweetened beverages (continuous). Country-specific hazard ratios were pooled using random-effects meta-analysis.

## Table S1. Baseline characteristics of the EPIC-InterAct case-cohort

| **Characteristic** | **Subcohort**  **(N=10,087)** | **Type 2 diabetes cases***  **(N=7,498)** |
| --- | --- | --- |
| Sex (% men) | 37.1 | 49.6 |
| Age (years) | 52.4 (12.4) | 55.8 (10.0) |
| BMI (kg/m^2^) | 25.8 (5.3) | 29.5 (5.9) |
| Education (%) |  |  |
| None | 8.8 | 11.5 |
| Primary school completed | 31.9 | 40.4 |
| Technical/professional school | 24.1 | 24.7 |
| Secondary school | 14.3 | 10.3 |
| Longer education (incl. University deg.) | 20.9 | 13.0 |
| Physical activity (%) |  |  |
| inactive | 22.6 | 29.0 |
| Moderately inactive | 33.4 | 33.0 |
| Moderately active | 22.4 | 19.6 |
| active | 21.6 | 18.5 |
| Smoking (%) |  |  |
| never | 46.4 | 40.3 |
| former | 27.2 | 30.8 |
| current | 26.4 | 28.9 |
| Country (%) |  |  |
| France | 3.0 | 1.9 |
| Italy | 14.7 | 14.3 |
| Spain | 25.6 | 24.1 |
| UK | 8.9 | 9.0 |
| The Netherlands | 11.1 | 7.9 |
| Germany | 17.8 | 18.5 |
| Denmark | 18.8 | 24.4 |
| Plasma phosholipid fatty acids |  |  |
| Linoleic acid (%) | 22.7 (4.2) | 21.6 (4.1) |
| α-linolenic acid (%) | 0.27 (0.18) | 0.25 (0.17) |
| Dietary intake |  |  |
| Total energy (kcal/d) | 2133 (862) | 2163 (900) |
| Carbohydrate (% total energy) | 46.7 (9.2) | 46.5 (10.0) |
| Protein (% total energy) | 15.5 (3.2) | 15.7 (3.5) |
| MUFA (% total energy) | 13.1 (4.1) | 12.9 (4.0) |
| PUFA (% total energy) | 6.0 (2.3) | 5.9 (2.3) |
| Linoleic acid (% total energy) | 1.76 (1.52) | 1.82 (1.62) |
| α-linolenic acid (% total energy) | 0.16 (0.15) | 0.16 (0.17) |
| Fiber (g/d) | 22.9 (10.4) | 22.7 (11.1) |
| Vitamin C (g/d) | 105 (80.5) | 101 (81.3) |
| Alcohol among consumers (g/d) | 7.18 (20.1) | 7.07 (22.4) |
| Nuts (g/d) | 0.8 (3.6) | 0.7 (2.1) |
| Fruits (g/d) | 196 (223) | 187 (220) |
| Vegetables (g/d) | 163 (140) | 158 (142) |
| Coffee (g/d) | 225 (426) | 223 (448) |
| Tea (g/d) | 12.3 (238) | 6.6 (190) |
| Sugar sweetened beverages (g/d) | 2.7 (57.1) | 6.5 (85.7) |

Values are median (IQR) unless otherwise specified; * includes external plus internal cases

## Table S2. Baseline characteristics of the PREDIMED study

| **Characteristic** | **MedDiet+Nuts (N=500)** | **Control (N=443)** |
| --- | --- | --- |
| Sex (% men) | 43 | 40 |
| Age (years) | 66.4±5.8 | 67.6±6.2 |
| BMI (kg/m^2^) | 29.6±3.6 | 30.1±3.6 |
| Smoking (%) |  |  |
| never | 59 | 60 |
| former | 26 | 23 |
| current | 15 | 16 |
| Plasma phosholipid fatty acids |  |  |
| Linoleic acid (%) | 20.9±3.3 | 20.5±3.0 |
| α-linolenic acid (%) | 0.11±0.04 | 0.10±0.04 |
| Dietary intake |  |  |
| Total energy (kcal/d) | 2324±600 | 2251±596 |
| Carbohydrate (% total energy) | 41.4±6.8 | 42.7±7.1 |
| Protein (% total energy) | 16.4±2.5 | 16.5±2.8 |
| MUFA (% total energy) | 19.6±4.3 | 19.1±4.6 |
| PUFA (% total energy) | 6.4±1.9 | 6.0±1.9 |
| Linoleic acid (% total energy) | 5.3±1.7 | 5.0±1.8 |
| α-linolenic acid (% total energy) | 0.6±0.2 | 0.5±0.2 |
| Nuts (g/d) | 13.4±15.1 | 8.9±12.3 |

Values are median (IQR) unless otherwise specified

## Table S3. Partial Spearman correlations* between diet and plasma linoleic acid (LA) and α-linolenic acid (ALA), EPIC-InterAct subcohort

| Country | Plasma Phospholipid PUFA | Dietary PUFA intake | | | | |
| --- | --- | --- | --- | --- | --- | --- |
|  |  | LA |  |  | ALA |  |
|  |  | r | p-value |  | r | p-value |
| France | LA | 0.047 | 0.43 |  | 0.045 | 0.45 |
| n=307 | ALA | 0.096 | 0.11 |  | 0.072 | 0.23 |
|  |  |  |  |  |  |  |
| Italy | LA | 0.125 | <0.01 |  | 0.101 | <0.01 |
| n=1,479 | ALA | -0.034 | 0.20 |  | -0.001 | 0.96 |
|  |  |  |  |  |  |  |
| Spain | LA | 0.071 | <0.01 |  | 0.017 | 0.38 |
| n=2,577 | ALA | -0.016 | 0.42 |  | 0.003 | 0.89 |
|  |  |  |  |  |  |  |
| United Kingdom | LA | 0.161 | <0.01 |  | 0.171 | <0.01 |
| n=900 | ALA | -0.031 | 0.36 |  | -0.055 | 0.10 |
|  |  |  |  |  |  |  |
| The Netherlands | LA | 0.124 | <0.01 |  | 0.105 | <0.01 |
| n=1,122 | ALA | 0.031 | 0.31 |  | 0.025 | 0.40 |
|  |  |  |  |  |  |  |
| Germany | LA | 0.115 | <0.01 |  | 0.094 | <0.01 |
| n=1,801 | ALA | 0.018 | 0.44 |  | 0.055 | 0.02 |
|  |  |  |  |  |  |  |
| Denmark | LA | 0.129 | <0.01 |  | 0.135 | <0.01 |
| n=1,901 | ALA | 0.082 | <0.01 |  | 0.056 | 0.02 |
|  |  |  |  |  |  |  |
| All^†^ | LA | 0.113 | <0.01 |  | 0.097 | <0.01 |
| n=10,087 | ALA | 0.016 | 0.40 |  | 0.020 | 0.17 |

* adjusted for sex, age, BMI (continuous), smoking (never, former, or current), education (none, primary school, technical or professional school, secondary school, or higher education), physical activity index (inactive, moderately inactive, moderately active, or active), alcohol (none, >0–<6, 6–<12, 12–<24 and ≥24 g/d), intake of dietary fiber (continuous), vitamin C (continuous), total energy intake (continuous), carbohydrates (continuous), protein (continuous), mono-unsaturated fatty acids (continuous), consumption of coffee (continuous), tea (continuous), fruits (continuous), vegetables (continuous), and sugar-sweetened beverages (continuous);

^†^ meta-analysed with random effects model

## Table S4. Pooled HRs and 95% CIs for the association between dietary linoleic acid (LA) and α-linolenic acid (ALA) intakes and plasma biomarkers and type 2 diabetes by sex; EPIC-InterAct

|  | **HR (95% CI)** | |
| --- | --- | --- |
|  | **Women (n: 9,884)** | **Men (7,244)** |
| **PUFA intake (% of total energy intake)*** | | |
| LA | 1.10 (1.02; 1.19) | 1.06 (0.97; 1.16) |
| ALA | 1.06 (0.99; 1.13) | 1.05 (0.97; 1.13) |
|  | | |
| **PUFA plasma phospholipids (%)**^†^ | | |
| LA | 0.81 (0.77; 0.86) | 0.84 (0.79; 0.90) |
| ALA | 0.95 (0.89; 1.01) | 0.94 (0.89; 1.00) |

* HRs per 1 SD (per 1.5% of total energy intake from dietary LA / per 0.15% of total energy intake from dietary ALA); models stratified for age and adjusted for sex, center, BMI (continuous), smoking (never, former, or current), education (none, primary school, technical or professional school, secondary school, or higher education), physical activity index (inactive, moderately inactive, moderately active, or active), alcohol (none, >0–<6, 6–<12, 12–<24 and ≥24 g/d), intake of dietary fiber (continuous) and vitamin C (continuous), intake of total energy intake (continuous), carbohydrates, protein, mono-unsaturated fatty acids, as well as polyunsaturated fatty acids minus LA (for LA) or minus ALA (for ALA) (all expressed as E%), consumption of coffee (continuous), tea (continuous), fruits (continuous), vegetables (continuous), and sugar sweetened beverages (continuous)

^†^ HRs per 1 SD of LA/ALA biomarker concentration; models stratified for age and adjusted for sex, center, BMI (continuous), smoking (never, former, or current), education (none, primary school, technical or professional school, secondary school, or higher education), physical activity index (inactive, moderately inactive, moderately active, or active), alcohol (none, >0–<6, 6–<12, 12–<24 and ≥24 g/d), intake of dietary fiber (continuous) and vitamin C (continuous), intake of total energy intake (continuous), consumption of coffee (continuous), tea (continuous), fruits (continuous), vegetables (continuous), and sugar sweetened beverages (continuous), and plasma phospholipid ALA (for LA) and vice versa

## Table S5. Association of dietary linoleic acid (LA) and α-linolenic acid (ALA) intakes and plasma fatty acid biomarkers with incident type 2 diabetes after exclusion of cases identified within the first 2 years of follow-up; EPIC-InterAct study

| **Fatty acid** | **HR (95% CI)** |
| --- | --- |
| PUFA intake (% of total energy intake)* | |
| LA | 1.07 (1.02; 1.14) |
| ALA | 1.06 (1.01; 1.12) |
|  | |
| PUFA plasma phospholipids (%)^†^ | |
| LA | 0.82 (0.78; 0.85) |
| ALA | 0.93 (0.89; 0.98) |

* HRs per 1 SD of LA/ALA intake (per 1.5% of total energy intake from dietary LA / per 0.15% of total energy intake from dietary ALA) stratified by age and adjusted for sex, center, BMI (continuous), smoking (never, former, or current), education (none, primary school, technical or professional school, secondary school, or higher education), physical activity index (inactive, moderately inactive, moderately active, or active), alcohol (none, >0–<6, 6–<12, 12–<24 and ≥24 g/d), consumption of coffee (continuous), tea (continuous), fruits (continuous), vegetables (continuous), and sugar sweetened beverages (continuous), intake of dietary fiber (continuous) and vitamin C (continuous), intake of total energy intake (continuous), carbohydrates, protein, mono-unsaturated fatty acids, as well as polyunsaturated fatty acids minus LA (for LA) or minus ALA (for ALA) (all expressed as E%)

^†^ HRs per 1 SD of LA/ALA biomarker concentration stratified by age and adjusted for sex, center, BMI (continuous), smoking (never, former, or current), education (none, primary school, technical or professional school, secondary school, or higher education), physical activity index (inactive, moderately inactive, moderately active, or active), alcohol (none, >0–<6, 6–<12, 12–<24 and ≥24 g/d), intake of dietary fiber (continuous) and vitamin C (continuous), intake of total energy intake (continuous), consumption of coffee (continuous), tea (continuous), fruits (continuous), vegetables (continuous), and sugar-sweetened beverages (continuous)

## Table S6. Genotype distribution of *FADS1* rs174547 (T>C) by country, EPIC-InterAct subcohort

| **Country** | **N** | **Genotype** | | | | | |  | **Allele counts** | |  | **MAF** |
| --- | --- | --- | --- | --- | --- | --- | --- | --- | --- | --- | --- | --- |
|  |  | **CC** | | **CT** | | **TT** | |  | **C** | **T** |  | **C (%)** |
|  |  | **n** | **%** | **n** | **%** | **n** | **%** |  |  |  |  |  |
| France | 307 | 28 | 9.1 | 132 | 43.0 | 147 | 47.9 |  | 188 | 426 |  | 30.6 |
| Italy | 1,479 | 130 | 8.8 | 582 | 39.4 | 767 | 51.9 |  | 842 | 2,116 |  | 28.5 |
| Spain | 2,577 | 269 | 10.4 | 1,067 | 41.4 | 1,241 | 48.2 |  | 1,605 | 3,549 |  | 31.1 |
| United Kingdom | 900 | 107 | 11.9 | 425 | 47.2 | 368 | 40.9 |  | 639 | 1,161 |  | 35.5 |
| The Netherlands | 1,122 | 137 | 12.2 | 470 | 41.9 | 515 | 45.9 |  | 744 | 1,500 |  | 33.2 |
| Germany | 1,801 | 186 | 10.3 | 762 | 42.3 | 853 | 47.4 |  | 1,134 | 2,468 |  | 31.5 |
| Denmark | 1,901 | 204 | 10.7 | 859 | 45.2 | 838 | 44.1 |  | 1,267 | 2,535 |  | 33.3 |
| All | 10,087 | 1,061 | 10.5 | 4,297 | 42.6 | 4,729 | 46.9 |  | 6,419 | 13,755 |  | 31.8 |

MAF: Minor allele frequency

## Table S7. Association between consumption of nuts (in g/day) and plasma phospholipid fatty acids and interaction with *FADS1* rs174547, EPIC-InterAct random sub-cohort (n=10,087)

| **Fatty acid*** | **Beta^†^ (SE) per g increment in nut consumption, p-value** | | | |  | **P interaction** | |
| --- | --- | --- | --- | --- | --- | --- | --- |
|  |  | ***FADS1* rs174547 (T>C) genotype** | | |  | **additive**^‡^ | **recessive**^§^ |
|  | **All (n = 10,087)** | **CC (n = 1,061)** | **CT (n = 4,297)** | **TT (n = 4,729)** |  | **(n = 10,087)** | **(n = 10,087)** |
| LA | 0.0007 (0.0002), <.0001 | 0.0015 (0.0005), 0.0028 | 0.0005 (0.0003), 0.0519 | 0.0006 (0.0002), 0.0065 |  | 0.2875 | 0.2629 |
| ALA | 0.0000 (0.0004), 0.9476 | 0.0009 (0.0013), 0.5268 | 0.0003 (0.0007), 0.6584 | -0.0006 (0.0006), 0.3280 |  | 0.8883 | 0.9555 |
| GLA | -0.0012 (0.0008), 0.1454 | -0.0034 (0.0029), 0.2287 | -0.0003 (0.0013), 0.8152 | -0.0012 (0.0010), 0.2558 |  | 0.5421 | 0.1804 |
| DGLA | -0.0006 (0.0003), 0.0300 | -0.0010 (0.0010), 0.3368 | -0.0001 (0.0005), 0.7709 | -0.0009 (0.0004), 0.0172 |  | 0.4621 | 0.6837 |
| AA | -0.0006 (0.0002), 0.0074 | -0.0014 (0.0006), 0.0177 | -0.0007 (0.0003), 0.0157 | -0.0002 (0.0003), 0.4007 |  | 0.0304 | 0.2069 |

* Fatty acids were ln-transformed

^†^ adjusted for age, sex, center, BMI (continuous), smoking (never, former, or current), education (none, primary school, technical or professional school, secondary school, or higher education), physical activity index (inactive, moderately inactive, moderately active, or active), alcohol (none, >0–<6, 6–<12, 12–<24 and ≥24 g/d), intake of total energy intake (continuous), consumption of coffee (continuous), tea (continuous), fruits (continuous), vegetables (continuous), and sugar-sweetened beverages (continuous)

^‡^ considering multiplicative interaction between nut consumption and *FADS1* rs174547 genotype (coded per C allele, additive model (0,1,2))

^§^ CC versus CT+TT

AA - arachidonic acid, ALA - α-linolenic acid, DGLA - dihomo-γ-linolenic acid, GLA - γ-linolenic acid, LA - linoleic acid
